# Influence of Age on Ocular Biomechanical Properties in a Canine Glaucoma Model with *ADAMTS10* Mutation

**DOI:** 10.1371/journal.pone.0156466

**Published:** 2016-06-06

**Authors:** Joel R. Palko, Hugh J. Morris, Xueliang Pan, Christine D. Harman, Kristin L. Koehl, Kirk N. Gelatt, Caryn E. Plummer, András M. Komáromy, Jun Liu

**Affiliations:** 1 Department of Biomedical Engineering, Ohio State University, Columbus, Ohio, United States of America; 2 Department of Ophthalmology, Washington University, St. Louis, Missouri, United States of America; 3 Center for Biostatistics, Ohio State University, Columbus, OH, United States of America; 4 Department of Small Animal Clinical Sciences, Michigan State University, Lansing, Michigan, United States of America; 5 Department of Small Animal Clinical Sciences, University of Florida, Gainesville, Florida, United States of America; 6 Department of Clinical Studies, University of Pennsylvania, Philadelphia, Pennsylvania, United States of America; 7 Department of Ophthalmology, Ohio State University, Columbus, Ohio, United States of America; Cardiff University, UNITED KINGDOM

## Abstract

Soft tissue often displays marked age-associated stiffening. This study aims to investigate how age affects scleral biomechanical properties in a canine glaucoma model with *ADAMTS10* mutation, whose extracellular matrix is concomitantly influenced by the mutation and an increased mechanical load from an early age. Biomechanical data was acquired from *ADAMTS10-*mutant dogs (n = 10, 21 to 131 months) and normal dogs (n = 5, 69 to 113 months). Infusion testing was first performed in the whole globes to measure ocular rigidity. After infusion experiments, the corneas were immediately trephined to prepare scleral shells that were mounted on a pressurization chamber to measure strains in the posterior sclera using an inflation testing protocol. Dynamic viscoelastic mechanical testing was then performed on dissected posterior scleral strips and the data were combined with those reported earlier by our group from the same animal model (Palko et al, IOVS 2013). The association between age and scleral biomechanical properties was evaluated using multivariate linear regression. The relationships between scleral properties and the mean and last measured intraocular pressure (IOP) were also evaluated. Our results showed that age was positively associated with complex modulus (p<0.001) and negatively associated with loss tangent (p<0.001) in both the affected and the normal groups, suggesting an increased stiffness and decreased mechanical damping with age. The regression slopes were not different between the groups, although the complex modulus was significantly lower in the affected group (p = 0.041). The posterior circumferential tangential strain was negatively correlated with complex modulus (R = -0.744, p = 0.006) showing consistent mechanical evaluation between the testing methods. Normalized ocular rigidity was negatively correlated with the last IOP in the affected group (p = 0.003). Despite a mutation that affects the extracellular matrix and a chronic IOP elevation in the affected dogs, age-associated scleral stiffening and loss of mechanical damping were still prominent and had a similar rate of change as in the normal dogs.

## Introduction

Glaucoma is known to have a complex pathophysiology with multiple risk factors including age, genetics and intraocular pressure (IOP). The mechanical properties of the load-bearing tissues of the eye may play an important role in the IOP-glaucoma relationship, especially those of the peripapillary sclera and lamina cribrosa. Finite element modeling of the optic nerve head (ONH) has suggested that the peripapillary sclera structural and material properties could significantly influence the levels of stresses and strains of the lamina cribrosa.[1–3] For example, larger ONH deformations were predicted in eyes with a more compliant peripapillary sclera at a given level of IOP elevation, suggesting that a stiffer peripapillary sclera may be protective from glaucoma. On the contrary, our previous studies showed that the corneoscleral biomechanical properties can also impact the dynamic profile of IOP [4, 5] in that an overall more compliant shell is more capable of damping IOP spikes and protecting the eye from damaging IOP fluctuations. [6, 7]

Age is an important factor in understanding the relationship between IOP and glaucoma, as corneoscleral stiffness and incidence of glaucoma have all been shown to increase with age. Previous studies have investigated the age-associated changes in the thickness and mechanical properties of the corneoscleral shell of humans and various animal models. For example, a decreased peripapillary scleral thickness and decreased peripapillary scleral strains during inflation were found in humans and monkeys of older age.[8, 9] Using uniaxial strip testing, the age-associated stiffening was found from anterior to posterior sclera in the human eye.[10, 11] Increase in the overall ocular rigidity with age has also been demonstrated based on *in vivo* pressure-volume relationships in human eyes.[12] The increased mechanical stiffness of the corneoscleral shell with age is thought to be largely caused by the buildup of non-enzymatic collagen cross-linking from advanced glycation end-products (AGEs) which occurs in most soft collagenous tissues over time.[13, 14] How physiologic, age-related stiffening of ocular tissues may protect against or aggravate the development and progression of glaucomatous damage remains unclear.[15]

The effects of IOP elevations and glaucoma on scleral mechanical properties have also been investigated. In a monkey experimental glaucoma model, Girard et al. found that the posterior sclera stiffened with chronic IOP elevations.[16] When investigating the viscoelastic properties in monkey eyes subjected to short-term IOP elevations, Downs et al. found an increase in the equilibrium modulus in the IOP elevation group when compared to control eyes.[7] Coudrillier et al. found changes in posterior scleral creep in post-mortem human eyes with glaucoma.[8] These studies suggested a connection between elevated IOP and alterations in scleral biomechanics.

Glaucoma animal models, both experimental and natural, continue to play a crucial role in unraveling the cause and effect relationship between IOP, age, ocular biomechanics, and glaucoma risk. In this study, we used the *ADAMTS10*-mutant canine model, a clinically well-described and naturally occurring open-angle glaucoma (OAG) model, to investigate the influence of age and *ADAMTS10* mutation on scleral biomechanical properties in the affected animals, as compared to the normal controls that had neither the mutation nor IOP elevations.

This canine OAG is inherited as an autosomal recessive trait and has been linked to a variant (G661R missense mutation) of the *ADAMTS10* gene which reduces the ADAMTS10 protein’s half-life.[17, 18] The ADAMTS family is associated with the microfibril assembly and regulation of TGF_β_ in the extracellular matrix.[19–24] The affected dogs begin developing elevations in IOP around 9 to 12 months of age due to increased outflow resistance at the trabecular meshwork and in the uveoscleral pathway.[25–30] The process of glaucoma development in this dog model is similar to that of humans with reduction in axoplasmic flow of the optic neurons.[31] The optic nerve demyelinates early in the disease process with coinciding glial cell hypertrophy and hyperplasia.[32, 33] The axonal loss in the affected animals shows a predilection for damage to larger diameter axons (> 2 μm) in the central regions of the ONH during chronic increases in IOP. These morphologic optic nerve changes occur prior to ophthalmoscopic changes or clinically apparent visual field losses.[32, 33] Unlike mouse and rat models, the lamina cribrosa of dog is similarly well-developed to that of humans and is made up of at least 10 to 15 sheets of collagenous connective tissue.[32] Prior to IOP elevations no changes are seen in the architecture of the lamina cribrosa in the affected animals. However, in moderate glaucoma, these dogs develop clear changes in the laminar tissues with an interlaminar spacing decrease of roughly 60% (i.e., the spacing between the lamellae of the laminar cribrosa decreases from 25 μm to 10 μm) and reduced lamina cribrosa pore alignment and pore size that progresses to near obliteration of laminar pore spacing with severe glaucoma.[31] Similar changes are also seen in the experimental hypertensive dogs, indicating that these changes are secondary to IOP elevations and not a direct result of the *ADAMTS10* mutation.[34]

The intent of this study was to investigate how age affected the scleral mechanics in these *ADAMTS10* mutant dogs with chronic IOP increase. We expected that, similar to humans and other animal models, the sclera would stiffen with age in both affected and control animals. However, the extent of scleral stiffening in the affected dogs could be different from the normal dogs. Extracellular matrix remodeling in response to chronic IOP elevations might further speed up the stiffening; while the effect of the *ADAMTS10* mutation may act in the opposite direction, as the mutation impacts the extracellular matrix from an early age prior to glaucoma onset.[35] We hypothesized that age-associated stiffening in the affected dogs would be more pronounced than the normal dogs due to their simultaneous exposure to IOP elevations.

In this study, we performed a range of biomechanical testing protocols on the canine sclera to obtain a more comprehensive evaluation of the biomechanics. Full globe pressure-volume relationship was measured using infusion tests to evaluate the overall ocular rigidity of the entire corneoscleral shell. Full globe inflation was then performed in the same eyes to assess pressure-strain relationship of the posterior sclera under a physiologic loading configuration. In an attempt to combine the existing data previously obtained from the young, pre-glaucoma dogs, dynamic mechanical testing on scleral strips was completed in the older animals following the same protocol. [35]

## Materials and Methods

### Animals

All animal protocols were approved by the IACUC of University of Florida, University of Pennsylvania, and Michigan State University. Colonies of beagle-derived dogs carrying the G661R *ADAMTS10* mutation were developed and maintained at the three university sites. All animals included in this study were born, raised, and maintained under similar environmental conditions at the three university sites.

In order to determine the genotype, blood samples were collected from all dogs, DNA extracted (QIAmp DNA Blood Mini Kit; Qiagen, Valencia, CA, USA) and a 567-bp fragment PCR amplified that included exon 17 of the *ADAMTS10* gene with the GA mutation (corresponding to the G661R mutation in the *ADAMTS10* protein(18)) using these primers: 5’- CTCACCCAAGGAACCAAAGA -3’ (forward) and 5’- agggaatggggatctctcac -3’ (reverse). The PCR products were gel purified (QIAquick Gel Extraction Kit; Qiagen, Valencia, CA, USA) and sequenced to determine the dogs’ genotypes (normal/wild-type or homo-/heterozygous for GA mutation).

A total of 5 eyes from 5 normal dogs (age range 69 to 113 months) and 10 eyes from 10 dogs with an *ADAMTS10* mutation (age range 21 to 131 months) were collected immediately following euthanasia with an overdose of sodium pentobarbital (≥85 mg/kg) via intravenous injection (Table 1). The normal group consisted of unaffected dogs that were either free (n = 2) or carriers (n = 3) of the G661R missense mutation in the *ADAMTS10* gene. All animals underwent regular, detailed ophthalmic examinations prior to euthanasia, including indirect ophthalmoscopy (Keeler All Pupil II; Keeler Instruments, Broomall, PA, USA) with condensing lens (Pan Retinal 2.2 D; Volk Optical, Mentor, OH, USA), slitlamp biomicroscopy (Kowa SL-15; Kowa Optimed, Torrance, CA, USA), indirect gonioscopy (3- or 4-Mirror Goniolens; Volk Optical), and rebound tonometry (Tonovet; Icare, Vantaa, Finland). An applanation tonometer (Tono-Pen Vet; Reichert, Depew, NY, USA) was used on the dogs in our previous study.[35] Previous studies have shown that the Tono-Pen readings were not significantly different from the Tonovet readings for normotensive eyes.[36, 37] Since all IOPs in our previous study were within the range of 10–18 mmHg, these IOP data were combined in the analyses without further conversion. Tonometry was performed on most of the dogs throughout their lifespans (Table 1). Dogs affected with advanced OAG were treated with latanoprost ophthalmic solution, a prostaglandin analogue, as needed to address potentially painful pressure spikes >50 mmHg. Fundus photographs were taken with either a RetCam II (Clarity Medical Systems, Pleasanton, CA) or a Kowa RC-2 (Kowa Company, Tokyo, Japan).

**Table 1:** Demographics of tested dogs. Age, gender, genotype, IOPs and tissue collection are presented.

Dog Identification	Gender	Eye	Site^5^	*ADAMTS10*-Genotype^2^	Age (months)	Last IOP (mmHg)	Max IOP (mmHg)	Clinical Disease Stage (ONH atrophy)^4^
G68^1^	M	OD	M/P	affected	5.6	16.0	n/a	none
G70^1^	M	OD	M/P	affected	5.6	14.0	n/a	none
G66^1^	M	OD	M/P	affected	7.0	17.0	n/a	none
G67^1^	M	OD	M/P	affected	7.0	17.0	n/a	none
ISA	M	OS	F	affected	21.1	20.7	20.7	none
AUR	F	OS	M/P	affected	21.3	21.3	21.7	mild
G2^1^	M	OD	M/P	affected	38.4	17.0	31.4	moderate
ANG	M	OD	F	affected	60.1	32.3	45.0	advanced
HAR^3^	F	OD	F	affected	67.3	55.0	65.3	advanced
GRIF^3^	M	OD	F	affected	89.4	48.0	75.2	advanced
FLA^3^	M	OD	F	affected	94.9	67.0	84.6	advanced
FOR^3^	M	OD	F	affected	94.9	81.0	81.0	advanced
FRE^3^	M	OD	F	affected	94.9	50.0	77.0	advanced
AME	F	OD	F	affected	124.9	23.7	28.7	moderate
ZIG	M	OS	F	affected	130.6	18.7	31.0	moderate
G69^1^	M	OD	M/P	carrier	5.6	16.0	n/a	none
G71^1^	M	OD	M/P	carrier	5.6	13.0	n/a	none
G72^1^	F	OD	M/P	carrier	5.6	11.0	n/a	none
G73^1^	F	OD	M/P	carrier	5.6	14.0	n/a	none
G12^1^	M	OD	M/P	carrier	32.6	15.0	n/a	none
CHU	F	OD	M/P	normal	69.1	10.7	17.0	none
HER	F	OS	F	carrier	72.1	12.0	19.7	none
NAD	F	OD	F	carrier	83.6	13.0	19.0	none
LUC	F	OS	M/P	normal	92.3	8.7	n/a	none
BRI	F	OD	F	carrier	113.0	13.0	17.3	none

^1^Samples also included in Palko et al. 2013[35]

^2^*ADAMTS10*-genotype: affected = homozygous for G661R missense mutation; carrier = unaffected, heterozygous for G661R missense mutation; normal = does not carry G661R missense mutation. Both carrier and normal dogs were included in the “normal” group for analysis.

^3^Eyes were treated with latanoprost ophthalmic solution (prostaglandin analogue) as needed to address pressure spikes >50 mmHg

^4^Degree of ONH atrophy according to Fig 3.

^5^The sites where the animal lived are indicated as F: University of Florida or M/P: Michigan State University and University of Pennsylvania (these animals were first raised at University of Pennsylvania and later transferred to Michigan State University).

Abbreviations: M, male; F, female; OD, right eye; OS, left eye; IOP, intraocular pressure; ONH, optic nerve head.

Whole globes were shipped overnight to the Ohio State University in moist containers on ice using plastic tubes with wet gauze placed on both ends to maintain high humidity air. All mechanical experimentation was completed within 36 hours postmortem. Following measurements of the eyes’ dimensions with A-mode ultrasound, each globe was first subject to an infusion test to measure its overall ocular rigidity and an inflation test to measure the tangential and radial strains in the posterior sclera. The eyes were then dissected and dynamic mechanical analysis (DMA) and strain-controlled ramp testing were performed on strips from both the anterior and posterior sclera.

### Infusion Testing

The dimensions of the eyes were measured with A-mode ultrasound in immersion. The measurement protocol and system has been described previously.[5] Briefly, ultrasound echoes along the anterior-posterior, nasal-temporal, and superior-inferior directions of the eye were acquired. Assuming sound velocities of 1605 m/s (cornea and sclera), [38, 39] 1540 m/s (aqueous and vitreous humor) [40], and 1645 m/s (lens), [39, 40] the dimensions of the eyes were determined from the measurement of the time of flight of the ultrasound echoes. Because of the use of the radiofrequency data in the analysis of the time flight, the resolution for the measurement of distance is determined by the sampling rate of the radiofrequency data, which is about 1.5 μm in the present study (500 MHz sampling). The infusion experiment protocol has been described previously [5]. Briefly, the globes were placed on a custom holder and immersed in saline. A 20G needle was inserted into the posterior chamber of the eye for infusion of PBS using a programmable infusion pump (PhDUltra, Harvard Apparatus, MA) controlled by a customized LabVIEW program (LabVIEW, National Instruments, CA). Infusion of the posterior chamber has been shown to minimize the “washout” effect observed in non-human eyes.[41] A second 20G needle inserted through the cornea into the anterior chamber was connected to a pressure sensor (P75, Harvard Apparatus, MA) that recorded the continuous pressure data using the LabVIEW program. The eye was first infused using the programmable pump to establish a stable baseline IOP of 15 mmHg. After stabilizing at the baseline pressure for at least 10 minutes, the eye was then infused for one second with a flow rate of 15 μL/s to simulate fast physiological IOP elevations as explained previously [5]. The infusions were repeated twice, and the globes were allowed to equilibrate for at least 10 minutes between the infusions.

Ocular rigidity *K* was calculated using Friendenwald’s equation (Eq 1). Because of the strong negative correlation between ocular volume *V*_*o*_ and ocular rigidity *K*,[42] we also calculated the normalized ocular rigidity *k* as the product of *K* and *V*_*o*_ (Eq 2).

$$K=\frac{\ln(\frac{IOP}{IOP_{0}})}{V-V_{0}}$$ (1)

$$k=V_{0}K$$ (2)

### Inflation Testing

After infusion testing, the globes were trephined at the cornea and the scleral shells were prepared and mounted on custom pressure chambers. The scleral shell was first preconditioned with 10 cycles of pressurization from 5 to 45 mmHg. After equilibration at 5 mmHg for 15 minutes, IOP was gradually increased from 5 to 20 mmHg at steps of 0.5 mmHg and then to 20 to 30 in 2.5 mmHg steps and finally at 35, 40 and 45 mmHg with 10 seconds of equilibration time at each pressure step. Ultrasound radiofrequency data from the cross-sectional scans of the posterior sclera were acquired at each pressure step along the circumferential and meridional directions using a high-frequency ultrasound system (Vevo660, VisualSonics Inc., Toronto) with a 55-MHz transducer. All data were sampled at rate of 500 M samples per second (DP105; Acqiris, NY). The displacement field was calculated using an ultrasound speckle tracking algorithm described previously.[43] A least-square strain estimator [44] was used to calculate the strains in the axial (along the ultrasound beam) and lateral (perpendicular to ultrasound beam) directions and converted to the tangential and radial strains based on coordinate transform.[5]

### Uniaxial Strip Testing

**Fig 1:**
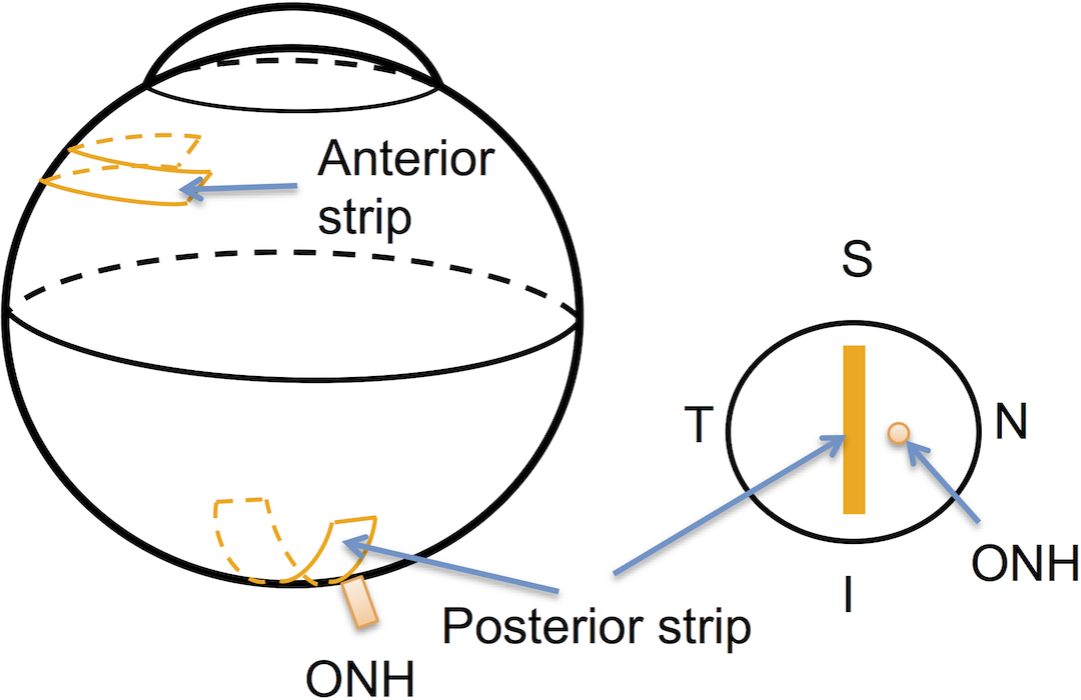
Schematics of the excision locations of the temporal anterior and posterior scleral strips. The posterior strip was cut adjacent to the temporal side of the ONH. S-Superior; I-Inferior; N-Nasal; T-Temporal.

After inflation testing, an anterior scleral strip and a posterior scleral strip adjacent to the ONH were excised from the temporal hemisphere of the globe (Fig 1). The strips were dissected using a parallel blade excision device described in previous studies.[35, 45]

The thickness and width of each strip was measured using high frequency B-mode ultrasound (Vevo660, VisualSonics, Toronto) as described previously.[45] The center frequency of the ultrasound imaging system is 55MHz, with an axial resolution of 30 μm and a lateral resolution of 62.5 μm. Briefly, three thickness measurements and one width measurement were taken at each of three cross-sectional scans along the central gage length. The averages of the nine total thickness measurements and three width measurements were used in further analysis. Prior to testing, the scleral strips were kept at 4°C and stored in phosphate buffered saline (PBS) solution. All mechanical testing was performed within 6 hours of strip excision.

Dynamic mechanical analysis (DMA) was first performed after sample mounting. DMA is one of the standard methods to determine the viscoelastic properties of a material.[46] A small amplitude, cyclic strain is used to induce a cyclic stress response. For a given sinusoidal strain, the resulting stress response will be sinusoidal if the applied strain is small enough so that the tissue can be approximated as linearly viscoelastic.[46, 47] For purely elastic material, the stress will be in phase with the applied strain; while a viscoelastic material will result in a phase lag δ. The ratio of the stress to strain along with the phase lag δ between the stress response and the applied strain are used to calculate the viscoelastic properties including the complex modulus E* and the loss tangent “tan(δ),” assuming linear viscoelasticity. The complex modulus E*, which can be thought as the overall resistance to deformation under dynamic loading (calculated as the ratio between the stress and the strain), has two components: the storage modulus (elastic component) and the loss modulus (viscous component). The loss tangent tan(δ), which is equal to the ratio of the loss modulus to storage modulus, represents the damping ability of the tissue.

In this study, DMA testing was performed using a Rheometrics System Analyzer (RSA III, TA Instruments, New Castle, DE) with a displacement resolution of 0.05 μm and a force resolution of 2 μN. Scleral strips were carefully mounted to ensure good alignment and prevent grip slippage. All samples were kept moist using a custom humidifying chamber and were tested at a temperature of approximately 37°C. The specimens were stretched from a relaxed state to a small load between 0.01 N and 0.02 N to flatten the curvature and ensure full contact between sample and grips. Preconditioning was performed for 90 seconds using cyclic triangular waves at a frequency of 0.1 Hz to a peak load amplitude of 0.1 N. The tissue was then allowed to equilibrate in the moist environment for 5 minutes.

The DMA testing was then performed with 12 cycles of a sinusoidal strain input at each increasing angular frequency of 0.1, 0.5, 1.0, 3.0, 5.0, and 10.0 Hz. Preliminary DMA testing was first conducted at various strain amplitudes on a group of canine posterior scleral tissue that was not used in the data collection of the present study. The preliminary testing showed that strain amplitudes below 0.25% generated a linear tissue response in canine sclera. Therefore, the strain amplitude of 0.15% was chosen for the present study. The evolution of the stress and strain over time was recorded for the last eight cycles for each frequency with a tissue rest time of 90 seconds between each frequency. Because dynamic responses are affected by the static preload, two preloads were used in the present study: 0.04 N and 0.1 N. These preloads aimed to generate tensile stresses in the tissue strips of the similar magnitudes as the hoop stresses in the canine sclera shell under either a “normal” (~15 mmHg) or “high” (~35 mmHg) IOP, based on Laplace law approximation using generic geometrical parameters of the eye (i.e., radius of curvature = 12 mm, and scleral thickness = 500 μm). Superimposing the small dynamic loads to these two preloads was intended to approximate the dynamic component of IOP (such as the ocular pulse) superimposed on either a “normal” or a “high” steady-state IOP. The dynamic testing was performed in the same manner at both preloads. The tissue was first brought to a preload level approximately 35% above the desired preload, allowed to relax for 5 min, and then manually adjusted to achieve the desired preload level.

Following all DMA testing, each strip was brought to the initial preload, allowed to equilibrate for 5 minutes, and then underwent a tensile ramp up to 3.5% strain at a strain rate of 0.1%/s. The engineering stress *σ* was calculated as the axial force divided by the original (i.e., unloaded) sample cross-sectional area (thickness × width). The strain *ε* was calculated as the displacement (i.e., change in grip distance during loading) divided by the original gauge length of the tissue sample after preloading. The nonlinear stress-strain data obtained from the tensile ramp tests was fit to an exponential model [48] in Eq (3) using the Levenberg-Marquardt least squares method:

$$\sigma =A(e^{B\varepsilon }-1)$$ (3)

The magnitude of A∙B represents an initial elastic modulus of the tissue while the B value represents the slope of change in the tissue’s tangent elastic modulus with increasing stress.

**Fig 2:**
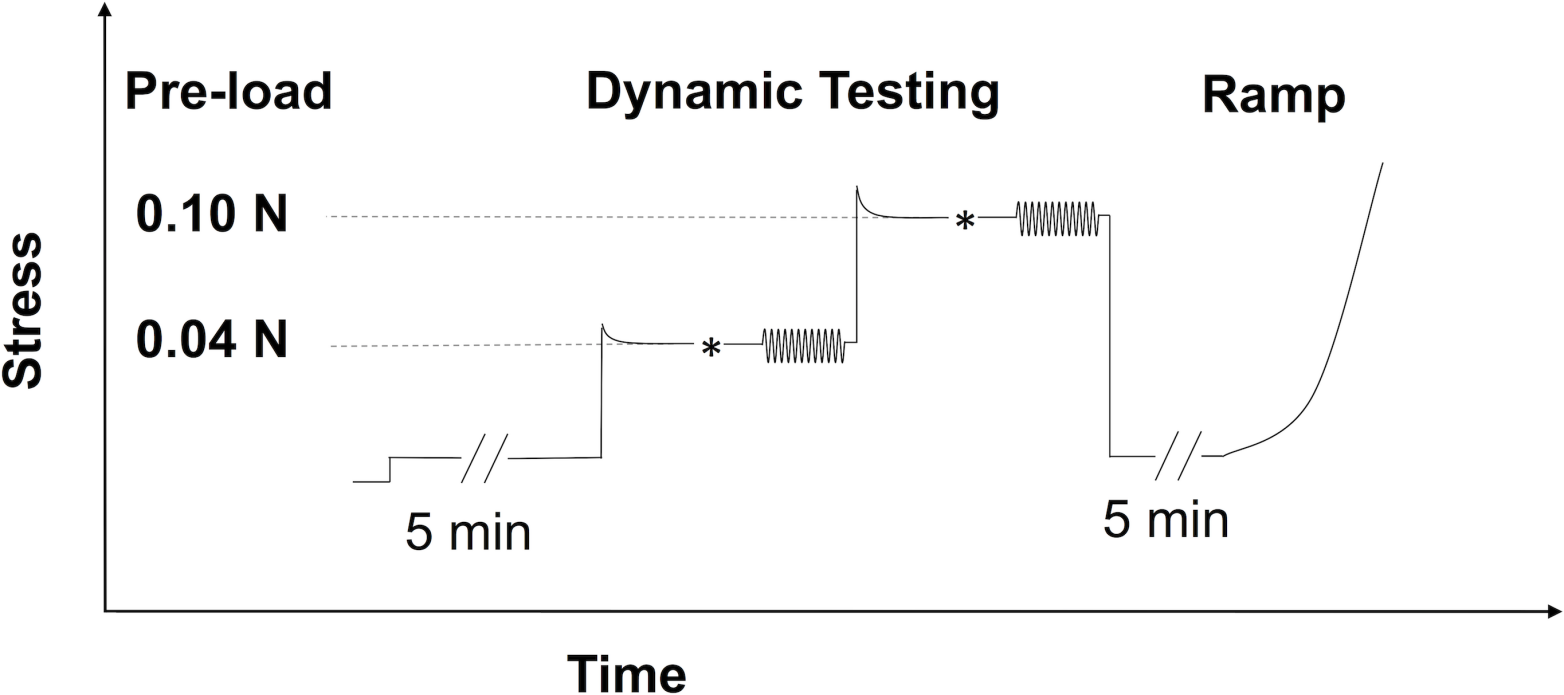
DMA and tensile testing protocols for each specimen strip. * indicated the small manual adjustments to fine tune the desired preload levels. Based on the predicted stress-relaxation that occurs in most soft tissue, the strip was first brought to a load 35% higher than the target pre-load (either 0.04 N or 0.10 N) prior to the manual adjustment. This is indicated by the initial stress overshoot in the figure)

An illustration of the overall testing protocol is presented (Fig 2).

### Statistical Analysis

Statistical analysis was performed using SAS 9.4 software package (SAS Institute Inc., Cary, NC). General linear regression models with the main effects and the interaction term of age and genotype were used to analyze the influence of age and genotype on the mechanical properties measured from uniaxial tests (i.e., complex modulus, loss tangent, B and A∙B), after combining data from the current study and those in our previous publication.[35] Similar linear regression models with the main effects of age and genotype were used to analyze the outcome from inflation tests (i.e., tangential and radial strains) and infusion tests (i.e., ocular rigidity). Pearson correlations were used to describe the associations between outcomes from different mechanical testing methods on the same tissue (i.e., tangential strains from inflation testing and complex modulus from uniaxial testing). Paired t-tests were used to compare the mechanical properties in the anterior and posterior sclera in the same eye.

## Results

**Fig 3:**
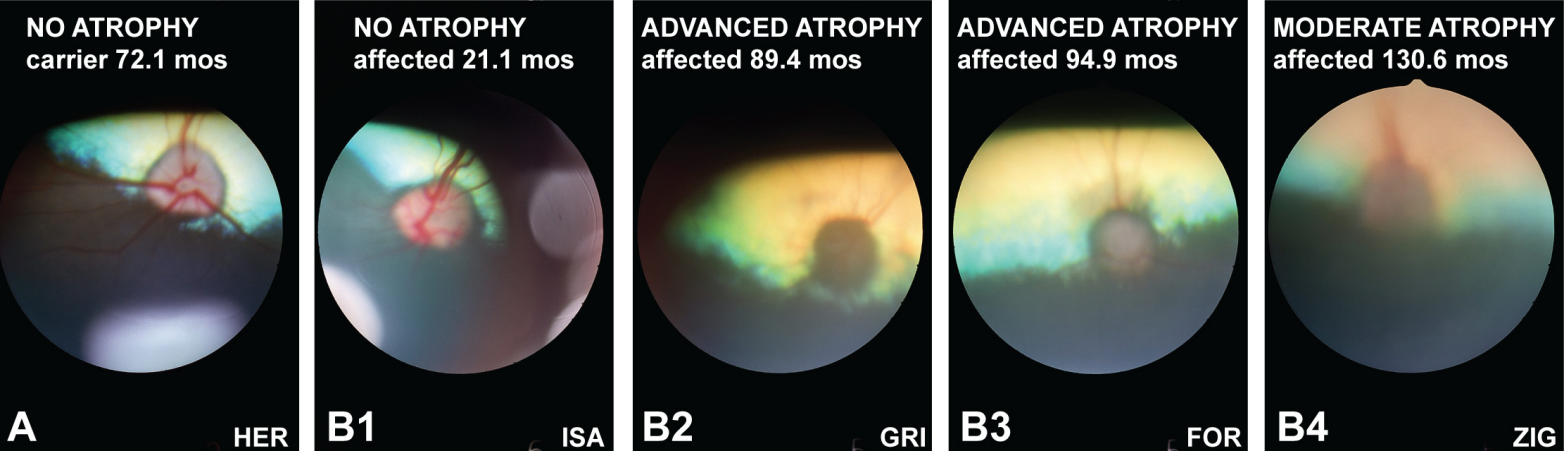
Fundus image of representative normal and affected dogs. The normal canine ONH appears pink and irregularly shaped due to myelination of the retinal ganglion cell axons as shown in this 72.1-month old carrier of the G661R *ADAMTS10* missense mutation (**A**). While the ONH still appears normal early in the disease process (**B1**, 21.1-month old affected dog), it becomes dark and round with advanced disease-related atrophy (**B2** and **B3**, 89.4- and 94.9-month affected dogs, respectively). With moderate atrophy, the ONH still appears slightly pink and irregularly shaped (**B4**). Because of secondary cornea and lens opacification with advanced glaucoma, the quality of the fundus images deteriorates and makes visualization of details such as cupping difficult. The identification of these representative dogs in the lower right corners of the images matches those in Table 1.

Clinical findings in *ADAMTS10*-mutant dogs prior to euthanasia were consistent with previously reported age- and disease-stage-related abnormalities;[18, 27, 28, 35, 49] these symptoms included slowly progressive rise of IOP with resulting optic nerve atrophy/cupping and associated vision loss, buphthalmia, ectopia lentis, secondary cataract formation and corneal edema. The iridocorneal angles appeared normal and open until late in the disease process.[27, 49] For the purpose of this study, clinical disease severity was graded according to the degree of ONH atrophy (Table 1; Fig 3). In addition to mutant dogs with classic disease phenotype and gradual IOP increase by 20 months of age and moderate to advanced ONH atrophy due to severely elevated IOP by 38–60 months, our study also included two affected dogs (ZIG and AME, Table 1) with a slower disease progression; these animals were still visual at >120 months of age with IOPs ≤31 mmHg.[49]

The primary outcome of biomechanical testing in this study was obtained from DMA and ramp tests on the posterior scleral strips. Because these same tests were also performed in our previous study on very young, pre-glaucomatous animals (less than 1 years old),[35] we were able to combine the two data sets to evaluate the age-associated trends and genotype comparisons. Table 2 summarizes the age and ocular dimensions in the affected and the normal groups.

**Table 2:** Age and ocular dimensions in the affected and normal groups. Results from the present study and our previous published data [35] are presented. NT: nasal-temporal; SI: superior-inferior.

Animal	Study	N	Age (months)	Axial Length (mm)	NT length (mm)	SI Length (mm)
**Affected**	**Previous**	5	14.5 ± 14.4	21.5 ± 1.0	21.74 ± 0.9	21.3 ± 0.9
	**Present**	10	81.3 ± 14.4	26.4 ± 2.4	22.9 ± 1.3	22.6 ± 1.5
** **	**Combined**	15	57.5 ± 45.3	24.9 ± 3.2	22.6 ± 1.3	22.3 ± 1.4
**Normal**	**Previous**	5	12.3 ± 12.1	20.7 ± 1.4	21.9 ± 0.5	26.4 ± 2.4
	**Present**	5	86.0 ±17.7	22.4 ± 2.1	21.5 ± 1.4	21.6 ± 0.5
** **	**Combined**	10	48.5 ± 42.0	21.5 ± 1.9	21.5 ± 1.1	21.1 ± 1.0

**Fig 4:**
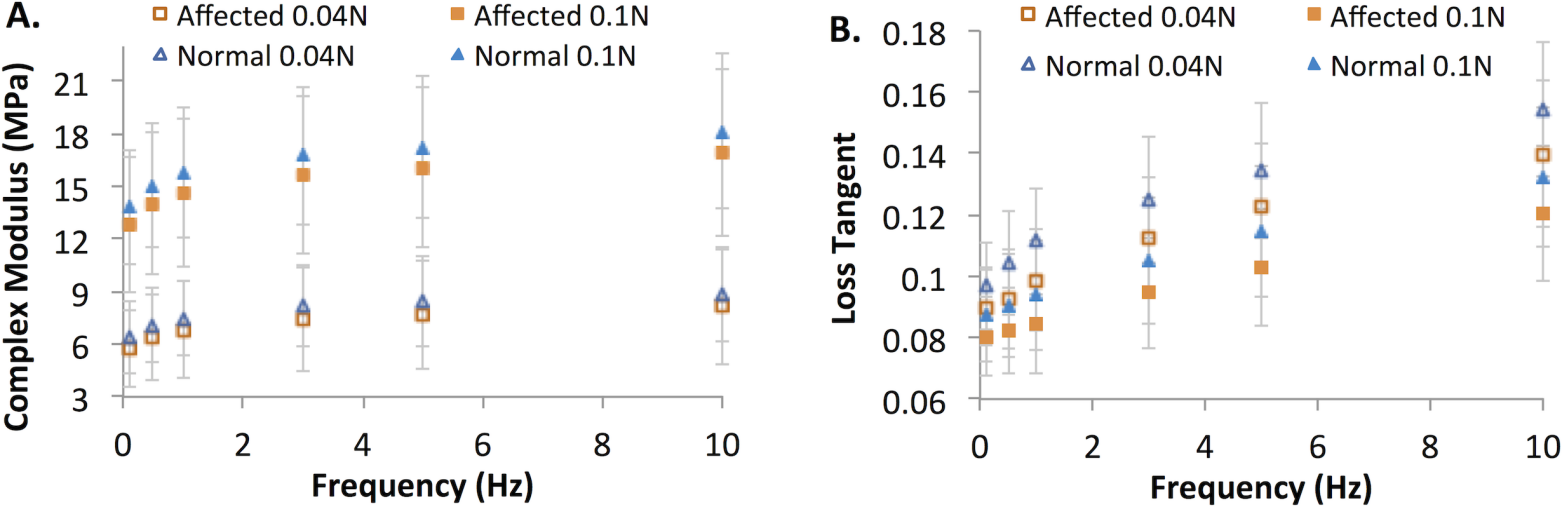
DMA parameters of the posterior sclera. Specimens from the affected (n = 15, squares) and normal (n = 10, triangles) groups were measured at six frequencies and two preloads (solid markers: preload at 0.1 N; open markers: preload at 0.04 N). **A:** Complex Modulus; **B:** Loss Tangent.

Fig 4 shows the complex modulus and loss tangent for the affected and normal groups measured at two different preload levels and six different frequencies (n = 15 for affected and n = 10 for normal). Preload and frequency had a statistically significant influence on the dynamic mechanical properties in both groups (all p < 0.001, linear mixed models), a similar outcome previously reported in the pre-glaucomatous young eyes.[35] The complex modulus increased at the larger preload and at higher frequencies. The loss tangent was greater at the lower preload and at higher frequencies. Despite the frequency and pre-load dependence, it can be observed from Fig 4 that the contrast between the affected and the normal groups was consistent across preloads and frequencies.

**Fig 5:**
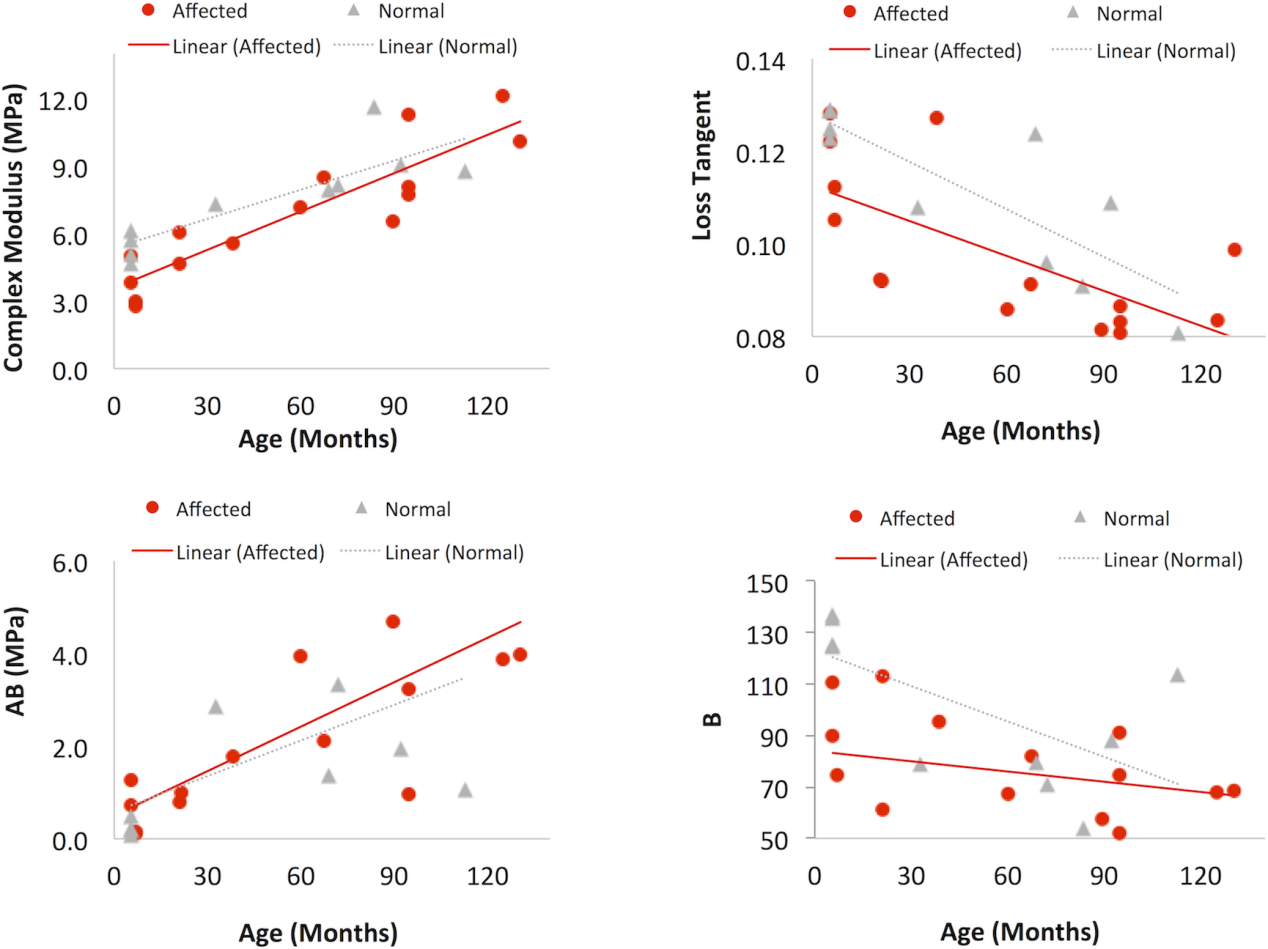
Age-associated changes in the posterior sclera. Affected: n = 15, red circles; normal: n = 10, gray triangles. Linear regression lines are shown for each group. **A:** complex modulus, **B:** loss tangent, **C:** initial tangent modulus A∙B, and **D**: slope of change B.

Linear regression models were applied to the DMA data at 1.0 Hz frequency and 0.04 N preloads to analyze age-associated changes in complex modulus and tan (δ) in the affected and normal groups, considering the interaction between age and genotype. The parameters from ramp tests (initial tangent modulus A∙B and slope of change B) were analyzed using similar linear regression models. Overall, complex modulus E^*^ was significantly increased with age (R = 0.857, p < 0.001). The rate of increase in E^*^ was not significantly different between groups (0.0568 MPa/month in the affected group and 0.0431 MPa/month in the normal group, p = 0.28). The loss tangent showed significant decrease with age (R = -0.716, p<0.001), and the rate of change in loss tangent was not significantly different in the two groups (p = 0.45). The initial tangent modulus A∙B showed significant increase with age (R = 0.668, p = 0.001) while B decreased with age (R = -0.44, p = 0.013). The thickness of the posterior sclera (measured from B-modes images of the dissected scleral strips) was negatively correlated with age (R = -0.827, p<0.001). The outcome of the linear regression analysis was not improved by considering the site where the animal lived as a covariate. These results are summarized in Fig 5.

The linear regression equations are as follows:

$$\text{The affected group}:\;\text{Complex Modulus}\;E^{*}(MPa)=3.574+0.05676\cdot Age(months)$$ (4)

$$\text{The control group}:\;\text{Complex Modulus}\;E^{*}(MPa)=5.361+0.04313\cdot Age(months)$$ (5)

The affected group:Loss Tangenttan(δ)=0.11253−0.000252⋅Age(months) (6)

The affected group:Loss Tangenttan(δ)=0.12803−0.000342⋅Age(months) (7)

The affected group:Initial Tangent ModulusA⋅B(MPa)=0.503+0.03196⋅Age(months) (8)

The control group:Initial Tangent ModulusA⋅B(MPa)=0.585+0.0256⋅Age(months) (9)

Considering age as a covariate, the generalized linear regression models were also used to compare the biomechanical properties in the affected and normal groups. Table 3 summarizes the comparison between posterior scleral thickness, DMA parameters (i.e., complex modulus and loss tangent), and ramp testing parameters (initial tangent modulus A∙B, and B) in the two groups. Complex modulus and B were statistically significant different (higher) in the normal group as compared to the affected group (p = 0.041and 0.014, respectively).

**Table 3:** Posterior scleral mechanical properties measured from uniaxial testing in the affected and normal groups. DMA data were measured at 0.04 N preload and 1 Hz. E^*^: complex modulus. (p-values were based on linear regression models considering age as a covariate).

Group	Thickness (mm)	E^*^ (MPa)	Loss tangent	A∙B (MPa)	B
**Affected (N = 15)**	0.493 ± 0.101	6.84 ± 2.85	0.098 ± 0.017	2.34 ± 1.92	76.3 ± 20.9
**Normal (N = 10)**	0.495 ± 0.090	7.45 ± 2.13	0.111 ± 0.017	1.83 ± 2.05	100.7 ± 29.9
**p-Value**	0.844	0.041	0.061	0.94	0.014

**Fig 6:**
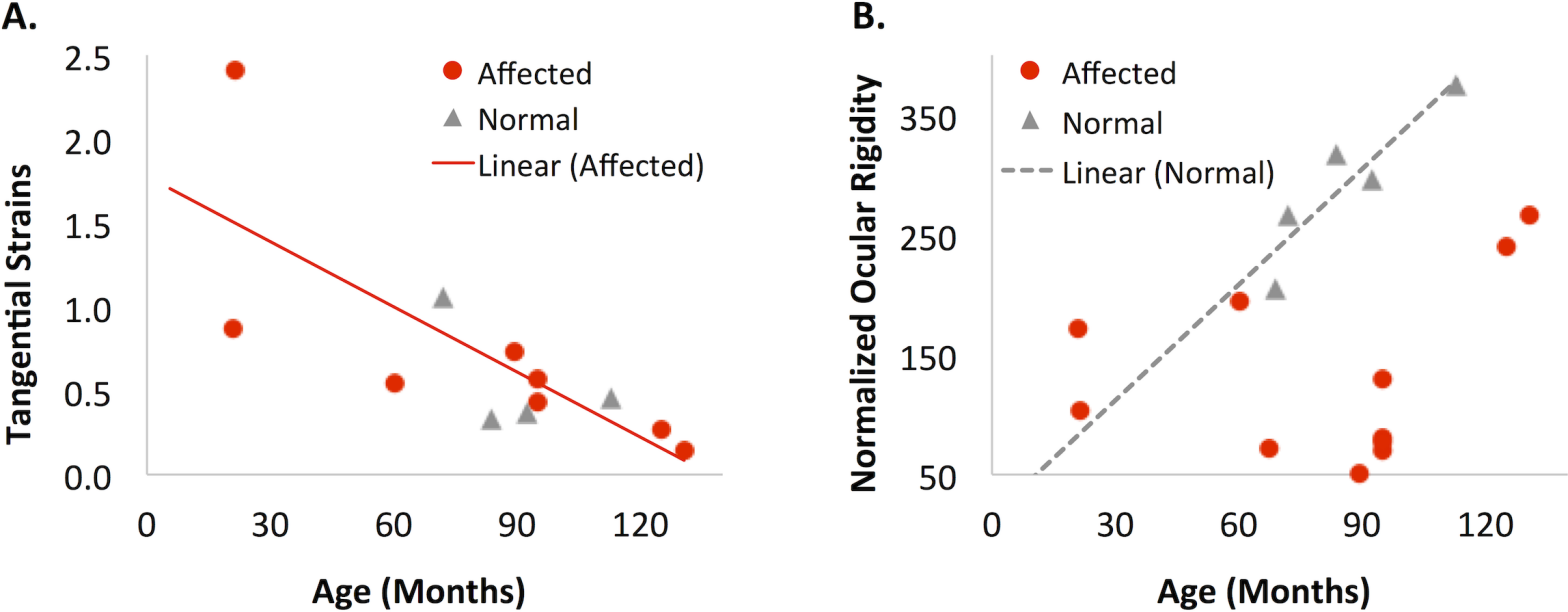
Relationship between age and biomechanical parameters from inflation and infusion testing. **A.** Tangential strains from the circumferential cross-section at 15 mmHg (Tc_15) in the posterior sclera decreased with age in the affected group (R = -0.764, p = 0.027, n = 8). **B.** Normalized ocular rigidity significantly increased with age in the normal group (R = 0.906, p = 0.034, n = 5). **Legend:** affected dogs (red circles); normal dogs (gray triangles).

Data from inflation testing were analyzed similarly with general linear regression models to evaluate the effect of age. Because of the relative small sample size especially in the normal group for this analysis, only the main effects of age and genotype were included. Based on the linear regression models, the posterior tangential strains from the circumferential cross-section (at 15 inflation pressure, Tc_15) had a significantly negative correlation with age in the affected group (p = 0.027, n = 8; Fig 6A). There was a similar trend in the normal group but the sample size (n = 4) was too small for sufficient statistical power. The tangential strains in the meridional cross-section (Tm_15) showed a similar trend of age-related decrease but it did not achieve statistical significance. None of the radial strains were associated with age. The strains at other inflation pressures were highly correlated with those at 15 mmHg in the same eye, similar to what was found before.[5] It is noted that although inflation testing was performed in 10 affected and 5 normal eyes, two eyes in the affected group and one eye in the normal group experienced experimental difficulties that rendered speckle tracking unsuccessful and therefore their strain data were unavailable. Based on linear regression models without the interaction term between age and genotype, the volume normalized ocular rigidity showed a trend of increasing with age, but it did not achieve statistical significance (p = 0.17). However, if the normal group was evaluated alone, there was a strong age-associated increase in normalized ocular rigidity (R = 0.91, p = 0.034). Scatterplots showing the relationships of posterior scleral strains and normalized ocular rigidity with age are presented (Fig 6B). Normalized ocular rigidity was higher in the normal group than the affected group (Fig 6B).

In addition to testing the main hypotheses described earlier, we explored answers to three important questions using the data acquired in the present study: [1] were anterior sclera biomechanical properties correlated with posterior sclera properties and were they different in the same eye? [2] was the mechanical testing outcome consistent among the different testing methods? and [3] was there an effect of IOP on ocular biomechanical properties?

**Anterior/Posterior sclera comparison.** Based on Pearson correlations, none of the anterior scleral biomechanical properties were correlated with the posterior scleral properties in the measured dogs. Based on linear regression models, none of the anterior scleral mechanical properties (e.g., complex modulus, loss tangent and initial tangent modulus A∙B, and B) were correlated with age. The anterior scleral thickness was 0.31 ± 0.030 mm in the affected group and 0.30 ± 0.054 mm in the normal group, and was significantly thinner than the posterior sclera in both groups (p<0.001 and p = 0.024, respectively; paired t-tests). The complex modulus and loss tangent of the anterior sclera at 1 Hz using 0.04 N preload were 12.51 ± 5.77 MPa and 0.089 ± 0.012 in the affected group and 17.17 ± 6.40 MPa and 0.090 ± 0.011 in the normal group; both marginally different from the posterior sclera (p = 0.052 and 0.059, respectively). The mean A∙B value for the anterior sclera was 9.42 ± 4.46 MPa in the normal group, significantly higher than the posterior sclera in the same eye (p = 0.045). The mean A∙B value for the anterior sclera was 5.82 ± 4.96 MPa in the affected group, not significantly different than the posterior sclera (p = 0.15). The mean B value for the anterior sclera was 56.8 ± 14.8 in the normal group, significantly higher than the posterior sclera (p = 0.02); the mean B value was 67.6 ± 17.5 in the affected group, not different from the posterior sclera (p = 0.40).

**Fig 7:**
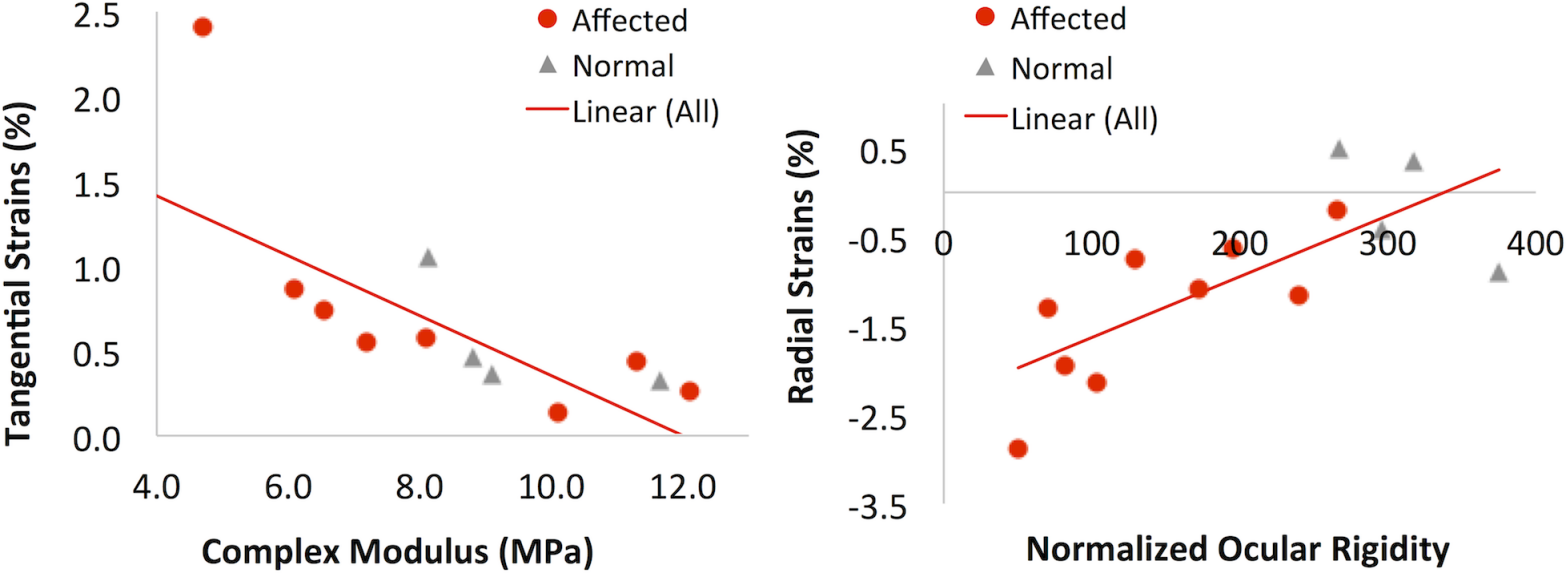
Different mechanical testing methods (uniaxial, inflation, and infusion) performed on the same tissue (posterior sclera) yielded consistent/correlative outcome. **A:** The tangential strains (Tc_15) from inflation testing were negatively correlated with the complex modulus from uniaxial testing; **B:** The radial strains (Rc_15, negative values suggesting compression) from inflation testing were positively correlated with the normalized ocular rigidity.

**Comparison between testing methods.** Comparing the outcome of the uniaxial strip testing and inflation testing, both performed on the posterior sclera in the present study, we found that the tangential strains (at 15 mmHg) in the circumferential cross-section (Tc_15) from inflation testing were strongly negatively correlated with the complex modulus: R = -0.744, p = 0.006 (n = 12, all eyes that had both data available, Fig 7A). The tangential strains in the meridional cross-section (Tm_15) also showed a trend of negative correlation with complex modulus but the trend did not reach statistical significance (R = -0.544, p = 0.104). There was also a strong positive correlation between the radial strains (Rc_15) in the circumferential cross-section at 15 mmHg and the volume normalized ocular rigidity: R = 0.78, p = 0.003 (n = 12, all eyes that had both data available, Fig 7B).

**Fig 8:**
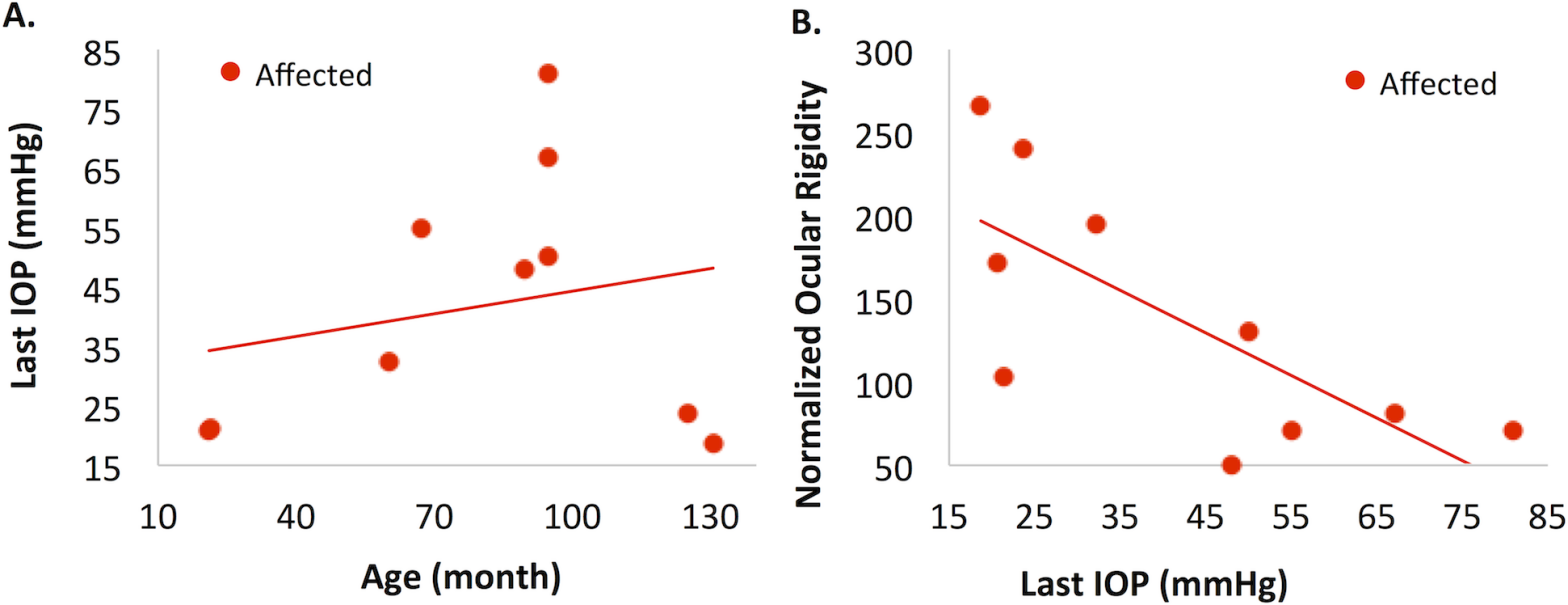
Relationship between IOP with age and ocular rigidity in the affected group. **A:** Last IOP was significantly positively associated with age in the affected group (p = 0.037). **B:** Last IOP was significantly negatively associated with normalized ocular rigidity (p = 0.003), but not significantly associated with any of the scleral biomechanical properties in the affected group.

**Influence of IOP.** We explored if IOP experienced by the animals, especially in the affected group, had an influence on their scleral biomechanical properties as well as the normalized ocular rigidity. Even though IOP was sporadically measured through the animals’ lives, only the IOP measured prior to euthanasia (last IOP) was consistently measured in all eyes. The last IOP was found to significantly positively correlate with age in the affected group (R = 0.54, p = 0.037 Fig 8A), but not in the normal group (p = 0.18). Although the last IOP appeared to be correlated with complex modulus and loss tangent in the affected group, linear regression models showed that those associations were not significant after considering the effect of age. However, there was a significant negative correlation between normalized ocular rigidity and last IOP, even after considering the age effect (p = 0.003; Fig 8B). The linear regression equations are:

Normalized ocular rigidity=181.1+0.995⋅Age(months)−2.940⋅LastIOP(mmHg) (10)

## Discussion

This study investigated the influence of age and genotype on the scleral biomechanical properties, as well as the overall ocular rigidity, in a canine glaucoma model. The primary outcomes are as follows: Age was associated with significant stiffening of the posterior sclera in both the normal and affected animals and the rate of stiffening increase with age was similar in both groups. The normalized ocular rigidity was not associated with age in the affected group but significantly increased with age in the normal group. The affected group had significantly lower complex modulus and mechanical constant B in the posterior sclera, as well as the normalized ocular rigidity, as compared to the normal group.

We observed a strong influence of age on the posterior scleral mechanical properties in both the affected and the normal dogs (Fig 5): the posterior sclera complex modulus and initial tangent modulus A∙B increased significantly with age, while loss tangent and B decreased significantly with age. The overall trend and rate of change in each of these biomechanical properties showed no difference between the two groups. Despite a much more compliant posterior sclera initially at an age prior to significant IOP increase,[35] the affected dogs showed a pronounced age-associated posterior scleral stiffening as the normal dogs. It was surprising that the rate of age-associated stiffening was similar in the affected and normal dogs, considering that the affected dogs had the *ADAMTS10* mutation and were exposed to gradually increasing IOPs, which could have affected the scleral extracellular matrix.[16] This observation suggested that either age plays a predominant role, or these other factors’ influences were balanced out with each other in this canine model. It is noted that most of the affected dogs also had sporadic treatment of prostaglandin drugs when the IOP spiked high. Prostaglandin analogues have been shown to reduce anterior scleral collagen content and thus possibly affect the mechanical stiffness.[50] The effect of prostaglandin on posterior sclera mechanical properties is not known. Given the topical application of the drug, its effect on posterior sclera collagen and mechanics is expected to be smaller than that on the anterior sclera.

Another interesting aspect of the age-related changes was the decreased B and loss tangent, which suggested a reduced rate of increasing stiffness to pressure increases and a reduced mechanical damping in the ageing eye. Grytz et al found a similar reduced nonlinearity with age in human donor eyes [51]. Although the effects of such changes are unclear, both trends appeared to indicate a weaker capacity for handling occasional quick IOP rise. Future studies are needed to evaluate if these trends are also found in the human sclera and how they may be related to the age-associated glaucoma risk.

Based on the linear regression models with age as a covariate, the posterior sclera in the affected group had a significantly lower complex modulus compared to the normal (Table 3). The affected group also had a lower B-value, indicating a reduced rate of stiffness increase during IOP increase. A lower complex modulus and a lower B-value were also observed in the young, pre-glaucomatous eyes in our previous study,[35] suggesting that these genotype-associated biomechanical differences in the posterior sclera were not altered during ageing.

Inflation testing of the scleral shell, which did not require disruption of the tissue microstructure, showed a consistent mechanical evaluation as uniaxial testing in scleral strips in terms of the overall stiffness: a smaller tangential strain (i.e., a stiffer response) was strongly correlated with a higher complex modulus in the same eye (Fig 7A). Inflation testing also confirmed the age-associated scleral stiffening showing a significant decrease of tangential strains with age (Fig 6A). Coudrillier et al reported a decrease in posterior scleral tangential strains and thickness with age in human donor eyes but did not find a significant difference in tangential strains between glaucomatous and non-glaucomatous eyes.[8] Interestingly, these same trends were observed in our canine glaucoma model: the tangential strain and thickness in the posterior sclera decreased with age in the affected group (p = 0.027 and <0.001, respectively), and the tangential strain was not significantly different between the groups. The lack of statistically significant difference in tangential strain might be attributed to the small number of animals whose tangential strain data was available in the present study, and future studies are needed to confirm the observations comparing glaucomatous and normal eyes.

The normalized ocular rigidity was lower in the affected group as compared to the normal group (Fig 6B). This was consistent with the lower average complex modulus in both the posterior sclera and the anterior sclera of the affected dogs, although the cornea likely plays an important role in determining the canine ocular rigidity. The canine cornea occupies a larger portion of the corneoscleral shell (with a horizontal diameter of 16–18 mm depending on breeds [52]) than the human cornea (with an average cornea horizontal diameter of 11.7 mm [53]). It was observed that the corneas in the affected dogs were generally enlarged and the axial length of the affected dogs was also significantly longer (Table 2), making the total intraocular volume larger in the affected dogs (i.e., buphthalmia). These changes were likely secondary to the chronically increased IOP starting from a relatively young age in this canine model. The observed overall negative correlation between last IOP and normalized ocular rigidity (Fig 8B) in the affected group supports this speculation. It was also interesting to observe a correlation between normalized ocular rigidity and the radial strain but not the tangential strain (Fig 7B), with a smaller radial strain (less compressive) being correlated with a higher ocular rigidity. It appears that during the short-term IOP and intraocular volume increase such as in the infusing tests for measuring ocular rigidity, the compressive properties of the ocular shell could play a significant role in absorbing the increased ocular volume and determining the level of IOP increase at a given change of volume.

As expected, there was a positive correlation between last IOP and age in the affected group (Fig 8A), since IOP is known to gradually increase in this canine glaucoma model. Because of the association between IOP and age, the age-related biomechanical changes in the affected group could be confounded by the increasing IOP. We thus developed linear regression models with both age and IOP as covariates, which showed that age remained a significant predictor (p<0.001) of the posterior scleral complex modulus in the affected group but IOP was not (p = 0.52). This again suggested that age was likely the primary factor in influencing scleral biomechanics, while IOP’s association with scleral modulus was mostly explained by its correlation with age in this canine model. Interestingly, the last IOP was negatively associated with the normalized ocular rigidity, even when considering age as a covariate.

It has been observed in the past that some affected dogs have much slower pressure increase and slower glaucoma progression compared to their littermates of the same genotype.[49] The two oldest affected dogs (AME: 124.9 months old, and ZIG, 130.6 months old; Table 1) in the present study were two such cases with slow glaucoma progression. Little is known about why this subset of affected dogs were better off even though they had the same mutation and were raised in identical conditions. Our data suggested that these two affected dogs may have a different ocular shell biomechanical profile compared to other affected dogs. For example, their posterior scleral complex modulus was among the highest in all tested dogs, which was also comparable to normal dogs of similar age (Fig 5A). Their overall normalized ocular rigidity, however, was much lower than what would be predicted for normal dogs showing only a small increase from the younger affected dogs (Fig 6B). It would be interesting to investigate whether the biomechanical properties of the cornea and sclera are different at the onset of glaucoma in this subset of dogs and whether these properties contribute to their relatively reduced glaucomatous damage. The canine glaucoma model presents a unique opportunity for studying these intriguing relationships between ocular biomechanics and glaucoma susceptibility, and may give insights to potential therapeutic approaches targeting mechanical alterations of the ocular shell.

There were several limitations in this study. First, the sample size was relatively small for older dogs due to limited availability, especially for the normal group. Applying different mechanical testing on the same eye provided confirmative evidences regarding the influence of age on scleral mechanical properties and strengthened the credibility of the results. Second, strains during inflation were measured on cross-sectional planes of the sclera, which is subjective to loss of tracking when out-of-plane motion is significant. Poor tracking was observed in two affected and one normal eye and their inflation data were not included in the analyses. Nonetheless, the use of very small incremental pressure steps, particularly within the lower pressure range, ensured that the speckle patterns were highly correlated in the consecutively acquired ultrasound data in most experimental eyes. Third, IOP data were not sampled at a high frequency in most animals, which limits an accurate estimate of the total IOP exposure and fluctuations experienced over the animal’s lifetime. Future studies will record weekly diurnal IOPs to systematically analyze the influence of IOP and interactions between IOP and ocular biomechanics.

In conclusion, this study showed age-associated stiffening of the posterior sclera at similar rate of change in dogs with or without the *ADAMTS10* mutation. Our previous study reported a significantly lower posterior scleral modulus in the affected young dogs prior to glaucoma onset as compared to the normal animals. This contrast persisted during ageing, despite the fact that the affected dogs experienced chronic IOP elevations. Although the scleral modulus was not significantly correlated with IOP when age was considered as a covariate, the normalized ocular rigidity was significantly negatively correlated with the last IOP in the affected dogs, likely due to the altered biomechanical properties from the *ADAMTS10* mutation and the enlarged cornea in this canine glaucoma model.

## Supporting Information

S1 Table IOP and ocular dimension data in each tested animal. Mean IOP: average of all IOP readings throughout the animal’s life (IOP was not read at regular intervals); Weighted IOP: mean IOP in the last two years of the animals’ lives weighted by the interval between measurements (calculated for animals that had more than one IOP readings); AL–axial length; NT: nasal-temporal length; SI–superior-inferior length.
(DOCX)

S2 Table Biomechanical parameters from uniaxial testing on posterior scleral strips. DMA: Dynamic mechanical analysis at 1 Hz for 0.04N preload; Goodness of fit: the fit of experimental stress-strain data to the exponential model using Eq (3).
(DOCX)

S3 Table Biomechanical parameters from uniaxial testing on anterior scleral strips. DMA: Dynamic mechanical analysis at 1 Hz for 0.04N preload; Goodness of fit: the fit of experimental stress-strain data to the exponential model using Eq (3).
(DOCX)

S4 Table Infusion and inflation testing parameters. *k*: normalized ocular rigidity as defined in Eq (2); Eye volume: estimated from an ellipsoidal shape with the three diameters matching the axial length, nasal-temporal length, and superior-inferior length in each eye; Rc_15: radial strain in the posterior sclera at 15 mmHg from a baseline of 5 mmHg; Tc_15: tangential strain in the posterior sclera at 15 mmHg from a baseline of 5 mmHg. Missing radial or tangential strain data were due to poor speckle tracking.
(DOCX)
