# Supplementary material for: Influence of Age on Ocular Biomechanical Properties in a Canine Glaucoma Model with ADAMTS10 Mutation
Source: PLoS One. 2016 Jun 6;11(6):e0156466. doi: 10.1371/journal.pone.0156466 (PMC4894564; doi:10.1371/journal.pone.0156466)
Supplement: S1 Table — Mean IOP: average of all IOP readings throughout the animal’s life (IOP was not read at regular intervals); Weighted IOP: mean IOP in the last two years of the animals’ lives weighted by the interval between measurements (calculated for animals that had more than one IOP readings); AL–axial length; NT: nasal-temporal length; SI–superior-inferior length. (DOCX) [file pone.0156466.s001.docx]

**S1 Table: IOP and ocular dimension data in each tested animal**. Mean IOP: average of all IOP readings throughout the animal’s life (IOP was not read at regular intervals); Weighted IOP: mean IOP in the last two years of the animals’ lives weighted by the interval between measurements (calculated for animals that had more than one IOP readings); AL – axial length; NT: nasal-temporal length; SI – superior-inferior length.

| **Animal** | **Mean IOP (mmHg)** | **Weighted IOP (mmHg)** | **AL (mm)** | **NT (mm)** | **SI (mm)** |
| --- | --- | --- | --- | --- | --- |
| FLA | 42.1 | 36.2 | 28.3 | 25.4 | 24.8 |
| FOR | 40.2 | 38.0 | 29.0 | 22.7 | 22.7 |
| GRIF | 40.7 | 31.9 | 26.9 | 24.0 | 23.6 |
| HAR | 34.7 | 39.0 | 30.2 | 23.6 | 23.1 |
| FRE | 38.4 | 51.5 | 27.2 | 24.5 | 24.6 |
| ANG | 29.5 | 32.3 | 26.1 | 22.5 | 21.5 |
| AME | 23.3 | 23.0 | 22.5 | 21.6 | 20.5 |
| ZIG | 24.4 | 25.4 | 27.3 | 22.1 | 22.5 |
| ISA | 18.6 | 20.0 | 23.1 | 22.1 | 22.4 |
| AUR | 17.1 | 17.2 | 25.5 | 22.1 | 23.0 |
| G2 | - | - | 23.3 | 23.1 | 22.5 |
| G66 | - | - | 21.5 | 22.0 | 22.1 |
| G67 | - | - | 21.1 | 21.1 | 20.8 |
| G68 | - | - | 21.0 | 21.2 | 20.3 |
| G70 | - | - | 20.7 | 21.2 | 20.8 |
| BRI | 13.5 | 13.0 | 25.8 | 22.0 | 21.9 |
| NAD | 13.4 | 13.3 | 23.0 | 21.9 | 21.2 |
| HER | 15.0 | 12.0 | 20.8 | 22.0 | 21.9 |
| LUC | - | - | 21.1 | 22.5 | 22.0 |
| CHU | 13.4 | 13.6 | 21.1 | 19.1 | 19.5 |
| G12 | - | - | 23.1 | 23.0 | 22.8 |
| G69 | - | - | 20.3 | 21.1 | 20.6 |
| G71 | - | - | 20.9 | 21.1 | 20.9 |
| G72 | - | - | 20.0 | 21.1 | 20.2 |
| G73 | - | - | 19.3 | 20.9 | 20.1 |
