# Supplementary material for: Influence of Age on Ocular Biomechanical Properties in a Canine Glaucoma Model with ADAMTS10 Mutation
Source: PLoS One. 2016 Jun 6;11(6):e0156466. doi: 10.1371/journal.pone.0156466 (PMC4894564; doi:10.1371/journal.pone.0156466)
Supplement: S2 Table — DMA: Dynamic mechanical analysis at 1 Hz for 0.04N preload; Goodness of fit: the fit of experimental stress-strain data to the exponential model using Eq (3). (DOCX) [file pone.0156466.s002.docx]

**S2 Table: Biomechanical parameters from uniaxial testing on posterior scleral strips.** DMA: Dynamic mechanical analysis at 1 Hz for 0.04N preload; Goodness of fit: the fit of experimental stress-strain data to the exponential model using Equation (3).

| **Animal** | **Thickness (mm)** | **DMA** | | **Ramp** | | |
| --- | --- | --- | --- | --- | --- | --- |
|  |  | **Complex Modulus (MPa)** | **Loss Tangent** | **A (MPa)** | **B** | **Goodness of Fit** |
| FLA | 0.470 | 8.11 | 0.081 | 0.011 | 90.98 | 98.7 |
| FOR | 0.418 | 11.31 | 0.086 | 0.125 | 52.20 | 96.9 |
| GRIF | 0.515 | 6.54 | 0.082 | 0.082 | 57.38 | 98.3 |
| HAR | 0.362 | 8.49 | 0.091 | 0.026 | 81.80 | 94.3 |
| FRE | 0.438 | 7.75 | 0.083 | 0.043 | 74.54 | 95.9 |
| ANG | 0.460 | 7.20 | 0.086 | 0.059 | 67.27 | 97.8 |
| AME | 0.394 | 12.12 | 0.083 | 0.057 | 68.12 | 97.6 |
| ZIG | 0.352 | 10.10 | 0.099 | 0.058 | 68.36 | 94.9 |
| ISA | 0.486 | 6.09 | 0.092 | 0.013 | 61.50 | 98.9 |
| AUR | 0.456 | 4.69 | 0.092 | 0.009 | 113.07 | 94.9 |
| G2 | 0.496 | 5.55 | 0.127 | 0.019 | 95.67 | 98.8 |
| G66 | 0.664 | 3.02 | 0.112 | 0.003 | 38.69 | 98.8 |
| G67 | 0.651 | 2.80 | 0.105 | 0.002 | 74.58 | 99.1 |
| G68 | 0.603 | 4.99 | 0.128 | 0.014 | 90.03 | 98.1 |
| G70 | 0.628 | 3.85 | 0.122 | 0.007 | 110.79 | 98.7 |
| BRI | 0.370 | 8.80 | 0.081 | 0.009 | 113.45 | 96.7 |
| NAD | 0.360 | 11.65 | 0.091 | 0.125 | 53.66 | 96.6 |
| HER | 0.440 | 8.13 | 0.096 | 0.047 | 71.28 | 95.9 |
| LUC | 0.483 | 9.09 | 0.109 | 0.022 | 87.87 | 98.7 |
| CHU | 0.477 | 7.94 | 0.124 | 0.017 | 79.57 | 96.9 |
| G12 | 0.465 | 7.29 | 0.108 | 0.036 | 79.11 | 99.2 |
| G69 | 0.593 | 4.68 | 0.129 | 0.001 | 135.85 | 98.5 |
| G71 | 0.598 | 5.11 | 0.129 | 0.002 | 137.00 | 98.3 |
| G72 | 0.587 | 6.14 | 0.125 | 0.004 | 124.40 | 98.9 |
| G73 | 0.576 | 5.69 | 0.123 | 0.002 | 125.15 | 96.3 |
