# Supplementary material for: Influence of Age on Ocular Biomechanical Properties in a Canine Glaucoma Model with ADAMTS10 Mutation
Source: PLoS One. 2016 Jun 6;11(6):e0156466. doi: 10.1371/journal.pone.0156466 (PMC4894564; doi:10.1371/journal.pone.0156466)
Supplement: S3 Table — DMA: Dynamic mechanical analysis at 1 Hz for 0.04N preload; Goodness of fit: the fit of experimental stress-strain data to the exponential model using Eq (3). (DOCX) [file pone.0156466.s003.docx]

**S3 Table: Biomechanical parameters from uniaxial testing on anterior scleral strips.** DMA: Dynamic mechanical analysis at 1 Hz for 0.04N preload; Goodness of fit: the fit of experimental stress-strain data to the exponential model using Equation (3).

| **Animal** | **Thickness (mm)** | **DMA** | | **Ramp** | | |
| --- | --- | --- | --- | --- | --- | --- |
|  |  | **Complex Modulus (MPa)** | **Loss Tangent** | **A (MPa)** | **B** | **Goodness of Fit** |
| FLA | 0.318 | 11.55 | 0.08 | 0.036 | 89.51 | 98.9 |
| FOR | 0.332 | 7.92 | 0.08 | 0.017 | 83.36 | 94.8 |
| GRIF | 0.325 | 11.09 | 0.07 | 0.082 | 58.52 | 97.8 |
| HAR | 0.305 | 8.70 | 0.09 | 0.032 | 71.04 | 98.9 |
| FRE | 0.305 | 9.59 | 0.09 | 0.039 | 84.10 | 98.8 |
| ANG | 0.330 | 13.89 | 0.09 | 0.177 | 44.75 | 97.5 |
| AME | 0.269 | 24.94 | 0.07 | 0.345 | 46.23 | 94.4 |
| ZIG | 0.337 | 8.79 | 0.10 | 0.096 | 47.53 | 97.6 |
| ISA | 0.335 | 8.15 | 0.11 | 0.023 | 81.96 | 98.9 |
| AUR | 0.247 | 20.44 | 0.09 | 0.219 | 59.17 | 94.3 |
| BRI | 0.360 | 8.60 | 0.09 | 0.037 | 81.03 | 97.5 |
| NAD | 0.267 | 16.51 | 0.10 | 0.150 | 54.74 | 98.5 |
| HER | 0.239 | 15.44 | 0.09 | 0.249 | 44.17 | 99.1 |
| LUC | 0.351 | 18.98 | 0.10 | 0.163 | 58.37 | 97.9 |
| CHU | 0.277 | 26.33 | 0.07 | 0.334 | 45.88 | 98.5 |
