# Supplementary material for: Influence of Age on Ocular Biomechanical Properties in a Canine Glaucoma Model with ADAMTS10 Mutation
Source: PLoS One. 2016 Jun 6;11(6):e0156466. doi: 10.1371/journal.pone.0156466 (PMC4894564; doi:10.1371/journal.pone.0156466)
Supplement: S4 Table — k: normalized ocular rigidity as defined in Eq (2); Eye volume: estimated from an ellipsoidal shape with the three diameters matching the axial length, nasal-temporal length, and superior-inferior length in each eye; Rc_15: radial strain in the posterior sclera at 15 mmHg from a baseline of 5 mmHg; Tc_15: tangential strain in the posterior sclera at 15 mmHg from a baseline of 5 mmHg. Missing radial or tangential strain data were due to poor speckle tracking. (DOCX) [file pone.0156466.s004.docx]

**S4 Table: Infusion and inflation testing parameters.** *k*: normalized ocular rigidity as defined in Equation (2); Eye volume: estimated from an ellipsoidal shape with the three diameters matching the axial length, nasal-temporal length, and superior-inferior length in each eye; Rc_15: radial strain in the posterior sclera at 15 mmHg from a baseline of 5 mmHg; Tc_15: tangential strain in the posterior sclera at 15 mmHg from a baseline of 5 mmHg. Missing radial or tangential strain data were due to poor speckle tracking.

| **Animal** | *k* | Eye Volume (ml) | Rc_15 | Tc_15 |
| --- | --- | --- | --- | --- |
| FLA | 81.4 | 9.34 | -1.94 | 0.57 |
| FOR | 70.2 | 7.82 | -1.30 | 0.43 |
| GRIF | 50.3 | 7.98 | -2.89 | 0.73 |
| HAR | 71.1 | 8.62 | - | - |
| FRE | 129.9 | 8.58 | -0.74 | -0.31 |
| ANG | 194.7 | 6.63 | -0.63 | 0.55 |
| AME | 240.2 | 5.21 | -1.15 | 0.26 |
| ZIG | 265.6 | 7.08 | -0.19 | 0.14 |
| ISA | 172.0 | 5.96 | -1.10 | 0.87 |
| AUR | 103.8 | 6.80 | -2.14 | 2.41 |
| BRI | 375.1 | 6.51 | -0.89 | 0.46 |
| NAD | 317.5 | 5.59 | 0.36 | 0.33 |
| HER | 267.0 | 5.25 | 0.48 | 1.05 |
| LUC | 296.4 | 5.47 | -0.43 | 0.36 |
| CHU | 204.8 | 4.10 | - | - |
